# Method for a new risk assessment of urban water quality: IFN-SPA

**DOI:** 10.1016/j.mex.2021.101237

**Published:** 2021-01-22

**Authors:** Hai-Min Lyu, Qian Zheng, Shui-Long Shen, Annan Zhou

**Affiliations:** a MOE Key Laboratory of Intelligent Manufacturing Technology, Department of Civil and Environmental Engineering, College of Engineering, Shantou University, Shantou, Guangdong 515063, China; b Civil and Infrastructure Engineering Discipline, School of Engineering, Royal Melbourne Institute of Technology (RMIT), Victoria 3001, Australia; c State Key Laboratory of Internet of Things for Smart City, University of Macau, Macau S.A.R., China

**Keywords:** SPA, Water quality, Risk assessment

## Abstract

Water quality is one of the most essential factors to influence human daily life and environment health. Risk assessment of water quality has critical significance to sustainable development of human society and natural systems. Set pair analysis (SPA) methods are widely used in risk assessment, especially in water resources. The essence of SPA is to classify assessment samples consider the uncertainties exist in risk assessment system based on the viewpoints of unity, difference, and opposition. The existing SPA methods are classified into two types, including (i) original SPA and (ii) comprehensive SPA. Both the original and comprehensive SPA methods have the following limitations: (i) it is need to judge whether the assessment factor belongs to type I or type II; (ii) it is need to judge whether the assessment factor is positive or negative. This method article gives a detailed description of the application of the existing SPA method. The method article is a companion paper with the original article [1].

• Description of SPA methods.

• Application of SAP methods in risk assessment of water quality.

• Calculate the weights of assessment factors.

## Graphical abstract

**Figure:**
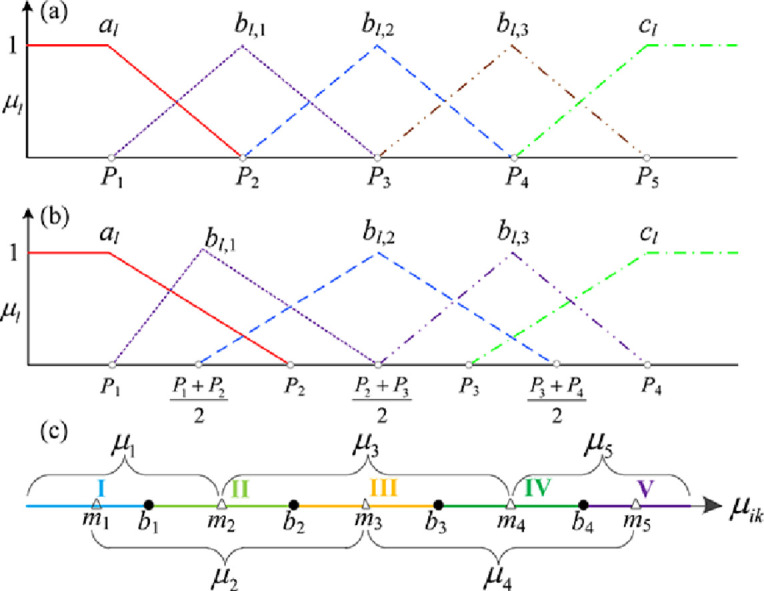

Specifications Table

**Table:** 

Subject Area	Engineering
More specific subject area	Water resource and environment
Method name	SPA application
Name and reference of original method	Risk assessment of set pair analysis [2,3].
Resource availability	*/*

## Introduction of SPA methods

### Original SPA

Suppose that set A and set B are two related sets, in which each set has *n* factors, e.g. A = (*a*_1_, *a*_2_, …… *a*_n_), where *x_l_* is the actual value of assessment factor and *n* is the number of assessment factors. Set B is assumed to be the assessment criteria. $B_{k}=b_{k}\left(k=1,2,3\cdots K\right)$, where *b*_k_ is the criterion value of level *k* and *K* is the classification level of the assessment sample. Set B = (*b*_1_, *b*_2_, …… *b*_n_). The connection degree between set A and set B can be described as below [2].

(1) $$\mu_{A↔B}=a+bi+cj$$

where $\mu_{A↔B}$ is the connection degree between set A and set B, *a* = identity degree, *b* = difference degree, and *c* = opposition degree, *a* + *b* + *c* = 1. The Eq. (1) can be referred to as a three-dimensional connection degree function (*K* = 3). If *K* = 5, Eq. (1) can be expressed as

(2) $$\mu_{A↔B}=a+b_{1}i_{1}+b_{2}i_{2}+b_{3}i_{3}+cj$$

where *a* + *b*_1_+*b*_2_+*b*_3_+*c* = 1. *b*_1_, *b*_2_, *b*_3_ are the difference components, which indicate the different levels of difference degree (such as mild difference, moderate difference, severe difference, etc.).

**Fig. 1:**
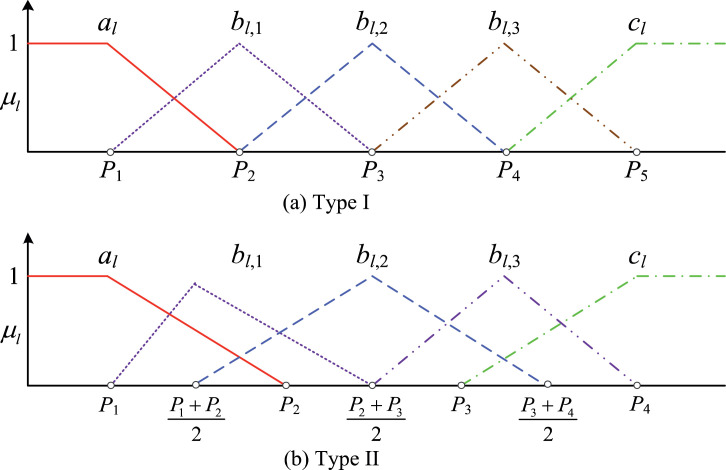
Collection degree membership of the original SPA (*K* = 5).

The original SPA method includes type I and type II. Table 1 lists the assessment criteria for types I and II. The risk level *K* is generally defined as three or five (*K* = 3 or *K* = 5). Fig. 1 shows the connection degree membership of type I and type II when *K* = 5. As shown in Fig. 1, type I uses a single value to express the risk level, whereas type II uses an interval value to express the risk level. For most of the decision makers, it is easier to assess the risk level using an interval value other than a single value. Therefore, type II is commonly used in risk assessment.

**Table 1:** Assessment criteria for the original SPA.

Type I					Type II				
1	2	…	*K*-1	*K*	1	2	…	*K*-1	*K*

*p*_1_	*p*_2_	…	*p*_k-1_	*p*_k_	<*p*_1_	*p*_1_~*p*_2_	…	*p*_k-2_~*p*_k-1_	>*p*_k-1_

The connection degree function of types I and II can be classified into positive factors and negative factors [3].

(I) In type I:

(1) for a positive factor, the connection degree function can be expressed using Eq. (3) [2],

(3) $$\mu_{l}=\left\{ \begin{array}{c} 1+0i_{1}+0i_{2}+\cdots +0i_{K-2}+0j\;\left(x_{l}\geq p_{1}\right)\\ \frac{x_{l}-p_{2}}{p_{1}-p_{2}}+\frac{p_{1}-x_{l}}{p_{1}-p_{2}}i_{1}+0i_{2}+\cdots +0i_{K-2}+0j\;\left(p_{2}\leq x_{l}<p_{1}\right)\\ 0+\frac{x_{l}-p_{3}}{p_{2}-p_{3}}i_{1}+\frac{p_{2}-x_{l}}{p_{2}-p_{3}}i_{2}+\cdots +0i_{K-2}+0j\;\left(p_{3}\leq x_{l}<p_{2}\right)\\ \cdots \\ 0+0i_{1}+0i_{2}+\cdots +\frac{x_{l}-p_{K}}{p_{K-1}-p_{K}}i_{K-2}+\frac{p_{k-1}-x_{l}}{p_{K-1}-p_{K}}j\;\left(p_{K}\leq x_{l}<p_{K-1}\right)\\ 0+0i_{1}+0i_{2}+\cdots +0i_{K-2}+1j\;\left(x_{l}<p_{K}\right) \end{array}\right.$$

(2) for a negative factor, the connection degree function can be expressed using Eq. (4)

(4) $$\mu_{l}=\left\{ \begin{array}{c} 1+0i_{1}+0i_{2}+\cdots +0i_{K-2}+0j\;\left(x_{l}\leq p_{1}\right)\\ \frac{p_{2}-x_{1}}{p_{2}-p_{1}}+\frac{x_{1}-p_{1}}{p_{2}-p_{1}}i_{1}+0i_{2}+\cdots +0i_{K-2}+0j\;\left(p_{1}<x_{l}\leq p_{2}\right)\\ 0+\frac{p_{3}-x_{l}}{p_{3}-p_{2}}i_{1}+\frac{x_{l}-p_{2}}{p_{3}-p_{2}}i_{2}+\cdots +0i_{K-2}+0j\;\left(p_{2}<x_{l}\leq p_{3}\right)\\ \cdots \\ 0+0i_{1}+0i_{2}+\cdots +\frac{p_{K}-x_{l}}{p_{K}-p_{K-1}}i_{K-2}+\frac{x_{l}-p_{K-1}}{p_{K}-p_{K-1}}j\;\left(p_{K-1}<x_{l}\leq p_{K}\right)\\ 0+0i_{1}+0i_{2}+\cdots +0i_{K-2}+1j\;\left(x_{l}>p_{K}\right) \end{array}\right.$$

Based on the single connection degree (*μ_i_*), the comprehensive connection degree ($\mu_{A↔B}$) of all assessment factors can be calibrated using Eq. (5) [2].

(5) $$\mu_{A↔B}=\sum_{l=1}^{n}w_{l}\mu_{l}=\sum_{l=1}^{n}w_{l}a_{l}+\sum_{l=1}^{n}w_{l}b_{l,1}i_{1}+\sum_{l=1}^{n}w_{l}b_{l,2}i_{2}+\cdots +\sum_{l=1}^{n}w_{l}b_{l,K-2}i_{K-2}+\sum_{l=1}^{n}w_{l}c_{l}j$$

where $\mu_{A↔B}$ is the comprehensive connection degree between set A (assessment sample) and set B (assessment criterion); *w_l_* is the weight of assessment factor; *μ_i_* is the single connection degree of factor *i*.

(II) In type II:

(1) for a positive factor, the connection degree function can be expressed using Eq. (6)

(6) $$\mu_{l}=\left\{ \begin{array}{c} 1+0i_{1}+0i_{2}+\cdots +0i_{K-2}+0j\;\left(x_{l}\leq p_{1}\right)\\ \frac{p_{1}+p_{2}-2x_{l}}{p_{2}-p_{1}}+\frac{2(x_{l}-p_{1})}{p_{2}-p_{1}}i_{1}+0i_{2}+\cdots +0i_{K-2}+0j\;\left(p_{1}<x_{l}\leq \frac{p_{1}+p_{2}}{2}\right)\\ 0+\frac{p_{2}+p_{3}-2x_{l}}{p_{3}-p_{1}}i_{1}+\frac{2x_{l}-p_{1}-p_{2}}{p_{3}-p_{1}}i_{2}+\cdots +0i_{K-2}+0j\;\left(\frac{p_{1}+p_{2}}{2}<x_{l}\leq \frac{p_{2}+p_{3}}{2}\right)\\ \cdots \\ 0+0i_{1}+\cdots +\frac{2(p_{K-1}-x_{l})}{p_{K-1}-p_{K-2}}i_{K-2}+\frac{2x_{l}-p_{K-2}-p_{K-1}}{p_{K-1}-p_{K-2}}j\;\left(\frac{p_{K-2}+p_{K-1}}{2}<x_{l}\leq p_{K-1}\right)\\ 0+0i_{1}+0i_{2}+\cdots +0i_{K-2}+1j\;\left(x_{l}>p_{K-1}\right) \end{array}\right.$$

When *K* = 5, the connection degree function can be expressed as below.

(7) $$\mu_{l}=\left\{ \begin{array}{c} 1+0i_{1}+0i_{2}+0i_{3}+0j\;\left(x_{l}\leq p_{1}\right)\\ \frac{p_{1}+p_{2}-2x_{l}}{p_{2}-p_{1}}+\frac{2(x_{l}-p_{1})}{p_{2}-p_{1}}i_{1}+0i_{2}+0i_{3}+0j\;\left(p_{1}<x_{l}\leq \frac{p_{1}+p_{2}}{2}\right)\\ 0+\frac{p_{2}+p_{3}-2x_{l}}{p_{3}-p_{1}}i_{1}+\frac{2x_{l}-p_{1}-p_{2}}{p_{3}-p_{1}}i_{2}+0i_{3}+0j\;\left(\frac{p_{1}+p_{2}}{2}<x_{l}\leq \frac{p_{2}+p_{3}}{2}\right)\\ 0+0i_{1}+\frac{2(p_{3}-x_{l})}{p_{3}-p_{2}}i_{2}+\frac{2x_{l}-p_{2}-p_{3}}{p_{3}-p_{2}}i_{3}+0j\;\left(\frac{p_{2}+p_{3}}{2}<x_{l}\leq \frac{p_{3}+p_{4}}{2}\right)\\ 0+0i_{1}+0i_{2}+\frac{2(p_{4}-x_{l})}{p_{4}-p_{3}}i_{3}+\frac{2x_{l}-p_{3}-p_{4}}{p_{4}-p_{3}}j\;\left(\frac{p_{3}+p_{4}}{2}<x_{l}\leq p_{4}\right)\\ 0+0i_{1}+0i_{2}+0i_{3}+1j\;\left(x_{l}>p_{4}\right) \end{array}\right.$$

where *μ_l_* is the single connection degree of assessment factor *l* and *x_l_* is the actual value of assessment factor *l*.

(2) for a negative factor, the connection degree function can be expressed using Eq. (8)

(8) $$\mu_{l}=\left\{ \begin{array}{c} 1+0i_{1}+0i_{2}+\cdots +0i_{K-2}+0j\;\left(x_{l}\geq p_{1}\right)\\ \frac{2x_{l}-p_{1}-p_{2}}{p_{1}-p_{2}}+\frac{2(p_{1}-x_{l})}{p_{1}-p_{2}}i_{1}+0i_{2}+\cdots +0i_{K-2}+0j\;\left(\frac{p_{1}+p_{2}}{2}\leq x_{l}<p_{1}\right)\\ 0+\frac{2x_{l}-p_{2}-p_{3}}{p_{1}-p_{3}}i_{1}+\frac{p_{1}+p_{2}-2x_{l}}{p_{1}-p_{3}}i_{2}+\cdots +0i_{K-2}+0j\;\left(\frac{p_{2}+p_{3}}{2}\leq x_{l}<\frac{p_{1}+p_{2}}{2}\right)\\ \cdots \\ 0+0i_{1}+\cdots +\frac{2(x_{l}-p_{K-1})}{p_{K-2}-p_{K-1}}i_{K-2}+\frac{p_{K-2}+p_{K-1}-2x_{l}}{p_{K-2}-p_{K-1}}j\;\left(p_{K-1}\leq x_{l}<\frac{p_{K-2}+p_{K-1}}{2}\right)\\ 0+0i_{1}+0i_{2}+\cdots +0i_{K-2}+1j\;\left(x_{l}<p_{K-1}\right) \end{array}\right.$$

When *K* = 5, the connection degree function can be expressed as below.

(9) $$\mu_{l}=\left\{ \begin{array}{c} 1+0i_{1}+0i_{2}+0i_{5}+0j\;\left(x_{l}\geq p_{1}\right)\\ \frac{2x_{l}-p_{1}-p_{2}}{p_{1}-p_{2}}+\frac{2(p_{1}-x_{l})}{p_{1}-p_{2}}i_{1}+0i_{2}+0i_{3}+0j\;\left(\frac{p_{1}+p_{2}}{2}\leq x_{l}<p_{1}\right)\\ 0+\frac{2x_{l}-p_{2}-p_{3}}{p_{1}-p_{3}}i_{1}+\frac{p_{1}+p_{2}-2x_{l}}{p_{1}-p_{3}}i_{2}+0i_{3}+0j\;\left(\frac{p_{2}+p_{3}}{2}\leq x_{l}<\frac{p_{1}+p_{2}}{2}\right)\\ 0+0i_{1}+\frac{2(x_{l}-p_{3})}{p_{2}-p_{3}}i_{2}+\frac{p_{2}+p_{3}-2x_{l}}{p_{2}-p_{3}}i_{3}+0j\;\left(\frac{p_{3}+p_{4}}{2}\leq x_{l}<\frac{p_{2}+p_{3}}{2}\right)\\ 0+0i_{1}+0i_{2}+\frac{2(x_{l}-p_{4})}{p_{3}-p_{4}}i_{3}+\frac{p_{3}+p_{4}-2x_{l}}{p_{3}-p_{4}}j\;\left(p_{4}\leq x_{l}<\frac{p_{3}+p_{4}}{2}\right)\\ 0+0i_{1}+0i_{2}+0i_{3}+1j\;\left(x_{l}<p_{4}\right) \end{array}\right.$$

where *μ_l_* is the single connection degree of assessment factor *l, x_l_* is the actual value of assessment factor *l*. For a five levels risk assessment system (*K* = 5), the connection degrees with the coefficients of *a, b*_1_, *b*_2_, *b*_3_ and *c* can be calibrated using Eq. (7) or Eq. (9).

### Comprehensive SPA

Eq. (10) shows the connection degree function of a comprehensive SPA [4].

(10) $$\mu_{pjk}=\left\{ \begin{array}{cc} 1-2\left|\frac{P_{j,k-1}-x_{pj}}{P_{j,k-1}-P_{j,k-2}}\right| & \left(x_{pj}\in k-1\right)\\ 1 & \left(x_{pj}\in k\right)\\ 1-2\left|\frac{x_{pj}-P_{j,k}}{P_{j,k+1}-P_{j,k}}\right| & (x_{pj}\in k+1)\;\\ -1 & (x_{pj}\in \text{other})\; \end{array}\right.$$

If the assessment factor is positive and *K* = 5, the connection degree function of *μ*_pjk_ can be expressed by using Eqs. (11)–(15).

(11) $$\mu_{j1}=\left\{ \begin{array}{c} 1,(x_{j}\leq p_{j1})\\ 1-2\cdot \left(x_{j}-p_{j1}\right)/\left(p_{j2}-p_{j1}\right),(p_{j1}<x_{j}\leq p_{j2})\\ -1,(x_{j}>p_{j2}) \end{array}\right.$$

(12) $$\mu_{j2}=\left\{ \begin{array}{c} 1-2\cdot \left(p_{j1}-x_{j}\right)/\left(p_{j1}-p_{j0}\right),\;\left(x_{j}\leq p_{j1}\right)\\ 1,\;(p_{j1}<x_{j}\leq p_{j2})\;\\ 1-2\cdot \left(x_{ij}-p_{j2}\right)/\left(p_{j3}-p_{j2}\right),\;\left(p_{j2}<x_{j}\leq p_{j3}\right)\\ -1,\;(x_{j}>p_{j3})\; \end{array}\right.$$

(13) $$\mu_{j3}=\left\{ \begin{array}{c} -1,\;(x_{j}\leq p_{j1})\\ 1-2\cdot \left(p_{j2}-x_{j}\right)/\left(p_{j2}-p_{j1}\right),\;\left(p_{j1}<x_{j}\leq p_{j2}\right)\;\\ 1,\;(p_{j2}<x_{j}\leq p_{j3})\\ 1-2\cdot \left(x_{j}-p_{j3}\right)/\left(p_{j4}-p_{j3}\right),\;\left(p_{j3}<x_{j}\leq p_{j4}\right)\\ -1,\;(x_{j}>p_{j4})\; \end{array}\right.$$

(14) $$\mu_{j4}=\left\{ \begin{array}{c} -1,(x_{ij}\leq p_{j2})\\ 1-2\cdot \left(p_{j3}-x_{j}\right)/\left(p_{j3}-p_{j2}\right),(p_{j2}<x_{j}\leq p_{j3})\\ 1,(p_{j3}<x_{j}\leq p_{j4})\\ 1-2\cdot \left(x_{j}-p_{j4}\right)/\left(p_{j5}-p_{j4}\right),(p_{j4}<x_{j}\leq p_{j5}) \end{array}\right.$$

(15) $$\mu_{j5}=\left\{ \begin{array}{c} -1,\;(x_{j}\leq p_{j3})\\ 1-2\cdot \left(p_{j4}-x_{j}\right)/\left(p_{j4}-p_{j3}\right),\;\left(p_{j3}<x_{j}\leq p_{j4}\right)\;\\ 1,\;(p_{j4}<x_{j}\leq p_{j5})\; \end{array}\right.$$

where *μ*_j1_, *μ*_j2_, *μ*_j3_, *μ*_j4_ and *μ*_j5_ are the connection degree with corresponding to risk level (*K* = 1, *K* = 2, *K* = 3, *K* = 4 and *K* = 5); *p*_j1_, *p*_j2_, *p*_j3_, *p*_j4_ and *p*_j5_ are the bounds of the criteria from level 1 to 5. If the assessment factor is positive and *K* = 5, the connection degree function of *μ*_pjk_ can be expressed using Eqs. (16)–(20).

(16) $$\mu_{j1}=\left\{ \begin{array}{c} 1,\;(x_{j}\geq p_{j1})\\ 1-2\cdot \left(x_{j}-p_{j1}\right)/\left(p_{j2}-p_{j1}\right),\;\left(p_{j1}>x_{j}\geq p_{j2}\right)\\ -1,\;(x_{j}<p_{j2})\; \end{array}\right.$$

(17) $$\mu_{j2}=\left\{ \begin{array}{c} 1-2\cdot \left(p_{j1}-x_{j}\right)/\left(p_{j1}-p_{j0}\right),\;\left(x_{j}\geq p_{j1}\right)\\ 1,\;(p_{j1}>x_{j}\geq p_{j2})\;\\ 1-2\cdot \left(x_{j}-p_{j2}\right)/\left(p_{j3}-p_{j2}\right),\;\left(p_{j2}>x_{j}\geq p_{j3}\right)\\ -1,\;(x_{j}<p_{j3})\; \end{array}\right.$$

(18) $$\mu_{j3}=\left\{ \begin{array}{c} -1,\;(x_{j}\geq p_{j1})\\ 1-2\cdot \left(p_{j2}-x_{j}\right)/\left(p_{j2}-p_{j1}\right),\;\left(p_{j1}>x_{j}\geq p_{j2}\right)\;\\ 1,\;(p_{j2}>x_{j}\geq p_{j3})\\ 1-2\cdot \left(x_{j}-p_{j3}\right)/\left(p_{j4}-p_{j3}\right),\;\left(p_{j3}>x_{j}\geq p_{j4}\right)\\ -1,\;(x_{j}<p_{j4})\; \end{array}\right.$$

(19) $$\mu_{j4}=\left\{ \begin{array}{c} -1,\;(x_{j}\geq p_{j2})\\ 1-2\cdot \left(p_{j3}-x_{i}\right)/\left(p_{j3}-p_{j2}\right),\;\left(p_{j2}>x_{j}\geq p_{j3}\right)\;\\ 1,\;(p_{j3}>x_{j}\geq p_{j4})\\ 1-2\cdot \left(x_{ij}-p_{j4}\right)/\left(p_{j5}-p_{j4}\right),\;\left(p_{j4}>x_{j}\geq p_{j5}\right)\; \end{array}\right.$$

(20) $$\mu_{j5}=\left\{ \begin{array}{cc} -1, & \left(x_{j}\geq p_{j3}\right)\\ 1-2\cdot \left(p_{j4}-x_{j}\right)/\left(p_{j4}-p_{j3}\right), & \left(p_{j3}>x_{j}\geq p_{j4}\right)\\ 1, & \left(p_{j4}>x_{j}\geq p_{j5}\right) \end{array}\right.$$

Based on the calibrated single connection degree (*μ*_pjk_), then the comprehensive connection degree (*μ*_pk_) can be obtained by using Eq. (21) as below:

(21) $$\mu_{pk}=\sum_{j=1}^{m}w_{j}\cdot \mu_{pjk}\;\left(1\leq j\leq n;\;1\leq k\leq K\right)$$

where *μ*_pk_ is the comprehensive connection degree of factor *p* to level *k* (−1 ≤ *μ*_pk_ ≤ 1); *μ*_pjk_ is the single connection degree of factor *p* to level *k*. When the value of *μ*_pk_ is closer to 1, the factor *p*_i_ has more possibility to belong the level *k*, while when the value of *μ*_pk_ is closer to −1, the factor *p*_i_ has more impossibility to belong the level *k*.

### Proposed IFN-SPA

**Fig. 2:**
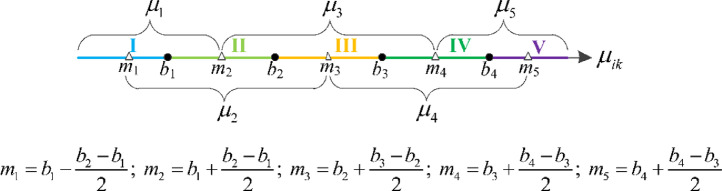
Interval-based fuzzy number SPA method.

Both the original and comprehensive SPA methods have the following limitations: (i) it is need to judge whether the assessment factor belongs to type I or type II; (ii) it is need to judge whether the assessment factor is positive or negative. This method article gives a detailed description of the application of the existing SPA method. Based on the review of the existing SPA methods, the IFN-SPA is proposed to modify the existing SPA methods by using the interval-based fuzzy numbers, in which an axis with interval criteria was adopted to express the connection degree of the assessment samples. The proposed IFN-SPA overcomes the limitations of the existing SPA methods. Fig. 2 draws the concept of the IFN-SPA. The detailed information of the proposed IFN-SPA method is presented in the companioned research article [1].

## Application of the method

The SPA method is combined with analytical hierarchy process (AHP) and others to assess risk [5], [6], [7], [8], [9]. The AHP is used to calibrate the weights of assessment factors. The AHP method uses pairwise comparison to establish the judgment matrix. Each element in the judgment matrix reflects the relative importance of assessment factors. The relative importance is quantified using number from 1 to 9 or their reciprocals [10]. Eq. (22) lists the judgment matrix (***A***) of the risk assessment of water quality in Shanghai. The detailed information of the assessment factors was presented in the original article [1].

(22) $$A={\left(a_{ij}\right)}_{n\times n}=\left(\begin{array}{ccccccccc} & R_{1} & R_{2} & R_{3} & R_{4} & R_{5} & R_{6} & R_{7} & R_{8}\\ R_{1} & 1 & 1 & 1 & 1 & 2 & 1.5 & 2 & 3\\ R_{2} & 1 & 1 & 1 & 1 & 1 & 2 & 2 & 3\\ R_{3} & 1 & 1 & 1 & 1.5 & 1 & 1.5 & 3 & 2\\ R_{4} & 1 & 1 & 0.667 & 1 & 2 & 2 & 1 & 3\\ R_{5} & 0.5 & 1 & 1 & 0.5 & 1 & 3 & 2 & 3\\ R_{6} & 0.667 & 0.5 & 0.667 & 0.5 & 0.333 & 1 & 5 & 6\\ R_{7} & 0.5 & 0.5 & 0.333 & 1 & 0.5 & 0.2 & 1 & 5\\ R_{8} & 0.333 & 0.333 & 0.5 & 0.333 & 0.333 & 0.167 & 1 & 1 \end{array}\right)$$

Based on the establishment of the judgment matrix (***A***), the weights (***w****_i_*) of the factors can be calculated from Eq. (23):

(23) $$w_{i}=\frac{M_{i}}{\sum_{i=1}^{n}M_{i}}$$

where $M_{i}=\sqrt[{n}]{\prod_{j=1}^{n}a_{ij}}$.

The judgment matrix (***A***) is reasonable, if the consistency ratio (*CR*) is less than 0.1. If the *CR* is greater than 0.1, then the judgment matrix is unreasonable and must be re-determined [11]. The value of *CR* can be calibrated using Eq. (24).

(24) $$CR=\frac{CI}{RI}$$

where $CI=\left(\lambda_{\max}-n\right)/\left(n-1\right)$ and *λ*_max_ is the largest eigenvalue of the judgment matrix; which can be calculated from Eq. (25). *RI* is the average random consistency index.

(25) $$\lambda_{\max}=\sum_{i=1}^{n}\frac{\sum_{j=1}^{n}a_{ij}w_{i}}{nw_{i}}$$

When *n* = 8, the *RI* = 1.41 [11], thus the value of *CR* = 0.0923 < 0.1.

Therefore, the judgment matrix (***A***) is reasonable. Then the weights of assessment factor can be calibrated as ***w***_i_ = (0.1632, 0.1551, 0.1574, 0.1474, 0.1372, 0.1152, 0.0764, 0.0481).

## Computational tool

The study used Excel sheets to calculate the evaluation of Shanghai water quality case for further illustration. The Excel include 10 sheets: IFN-SPA, AHP, R1 to R8 sheets, respectively. R1 to R8 sheets calculate the single connection degree (*μ_jk_*) of each safety risk level of water resource (level I to level V) of each district in Shanghai under the same assessment factor. It includes the field measured data (R_j_) of the water quality assessment factors for each district division, and the grading criteria (b_0_ to b_4_) for these assessment indicators. AHP sheet calculates the weight coefficient (*w_i_*) of eight assessment factors. It needs to input the comparative score of importance between two adjacent assessment factors. IFN-SPA sheet calculates the comprehensive connection degree under different levels. The calculation parameters of IFN-SPA sheet contain the values of single connection degree (*μ_jk_*) and the weight coefficient (*w_i_*). Therefore, the specific steps to use the calculation tool are as follows: first, input the measured data (R_j_) of the corresponding assessment factor into the orange area of sheet R1 to R8 to calculate the single connection (*μ_jk_*) and the results are shown in the blue area. Second, it needs input the score of importance into the orange area of AHP sheet to calculate the weights coefficient (*w_i_*) of assessment factors in blue area. Finally, it needs input the values of the single connection (*μ_jk_*) and the weight coefficient (*w_i_*) into the orange area of IFN-SPA sheet to calculate the comprehension connection degree (*µ*) and the results are shown in the red area of IFN-SPA sheet.

## Method validation

To verify the advantage of the proposed IFN-SPA method, both the existing SPA methods and the IFN-SPA method were used calibrate the connection degree of the assessment factors to the risk of water quality. Based on the weights of assessment factors calibrated from AHP method, the connection of each assessment samples can be obtained. Table 2 lists the connection degree calibrated from original SPA. Table 3 lists the connection degree calibrated from comprehensive SPA. Table 4 lists the connection degree calibrated from the proposed IFN-SPA. The detailed comparison and analyses of the assessed results can be found in the companioned research article [1].

**Table 2:** Connection degree calibrated from original SPA.

District	Pudong					Huangpu					Xuhui				
*μ_i_*	*a* (I)	*b*_1_ (II)	*b*_2_ (III)	*b*_2_ (IV)	*c* (V)	*a* (I)	*b*_1_ (II)	*b*_2_ (III)	*b*_2_ (IV)	*c* (V)	*a* (I)	*b*_1_ (II)	*b*_2_ (III)	*b*_2_ (IV)	*c* (V)
*μ*_PP_	0	0.9162	0.0838	0	0	0	0	0	0	1	0	0	−1.0782	2.0782	0
*μ*_GDPP_	0	0	0	0	1	0	0	0.5217	0.4783	0	0	0	−0.7935	1.7935	0
*μ*_IOUA_	0	0	−1.673	2.673	0	0	0.4155	0.5845	0	0	0	0	0	0.089	0.911
*μ*_BAR_	0	0.7188	0.2812	0	0	0	0	0	0	1	0	0	0	1.0467	−0.0467
*μ*_GAR_	0	0	0	0.5365	0.4635	1	0	0	0	0	0	0	0	0	1
*μ*_WRP_	0	0	0	0	1	0.1805	0.8195	0	0	0	−0.0893	1.0893	0	0	0
*μ*_WRUR_	0	0	0.8	0.2	0	0	0	0	0	1	0	0	0	1	0
*μ*_STA_	0	0	0	0	1	0	0	0	0.2	0.8	0	0	0	0	1
*μ*	0	0.2599	−0.1473	**0.5069**	0.3805	0.1574	0.1592	0.1722	0.0835	**0.4277**	−0.0103	0.1250	−0.2977	**0.8628**	0.3202
District	Changning					Jingan					Putuo				
*μ_i_*	*a* (I)	*b*_1_ (II)	*b*_2_ (III)	*b*_2_ (IV)	*c* (V)	*a* (I)	*b*_1_ (II)	*b*_2_ (III)	*b*_2_ (IV)	*c* (V)	*a* (I)	*b*_1_ (II)	*b*_2_ (III)	*b*_2_ (IV)	*c* (V)

*μ*_PP_	0	0	−0.6627	1.6627	0	0	0	0	0.1396	0.8604	0	0	0	0.8817	0.1183
*μ*_GDPP_	0	0	−0.9834	1.9834	0	0	0	0	0.4175	0.5843	0	0	−0.7954	1.7954	0
*μ*_IOUA_	0.078	0.922	0	0	0	0	0.684	0.316	0	0	0.148	0.852	0	0	0
*μ*_BAR_	0	0	−0.9434	1.9434	0	0	0	0	0	1	0	0	−0.984	1.984	0
*μ*_GAR_	0	0	0	0	1	0	0	−0.804	1.804	0	0	0	0	0.047	0.953
*μ*_WRP_	0	0.8205	0.1795	0	0	1	0	0	0	0	0	0.6568	0.3432	0	0
*μ*_WRUR_	0	0	−0.8	1.8	0	0	0	0	0	1	0	0	0	1	0
*μ*_STA_	0	0	0	0.4	0.6	0	0	0	0.2	0.8	0	0	0	0.6	0.4
*μ*	0.0122	0.2387	−0.4427	**1.0263**	0.1654	0.1147	0.1072	−0.0603	0.3433	**0.4953**	0.0232	0.2089	−0.2355	**0.8347**	0.1686
District	Hongkou					Yangpu					Minhang				
*μ_i_*	*a* (I)	*b*_1_ (II)	*b*_2_ (III)	*b*_2_ (IV)	*c* (V)	*a* (I)	*b*_1_ (II)	*b*_2_ (III)	*b*_2_ (IV)	*c* (V)	*a* (I)	*b*_1_ (II)	*b*_2_ (III)	*b*_2_ (IV)	*c* (V)

*μ*_PP_	0	0	0	0	1	0	0	−1.458	2.458	0	0	0.5615	0.4385	0	0
*μ*_GDPP_	0	0	0	0.1347	0.8653	0	0	−0.4534	1.4534	0	0	0	−0.9619	1.9619	0
*μ*_IOUA_	0.64	0.36	0	0	0	0	0	0	0	1	0	0	0	0.748	0.252
*μ*_BAR_	0	0	0	0	1	0	0	−0.5964	1.5964	0	0	0.3179	0.6821	0	0
*μ*_GAR_	0	0.578	0.422	0	0	0	0	0	0.5235	0.4765	0	0	−0.084	1.084	0
*μ*_WRP_	−0.9087	1.9087	0	0	0	0	0	−0.3595	1.3595	0	0	0	0	0.6865	0.3135
*μ*_WRUR_	0	0	0	0	1	0	0	0.8	0.2	1	0	0.5	0.5	0	0
*μ*_STA_	0	0	0	1	0	0	0	0.4	0.6	0	0.2	0.8	0	0	0
*μ*	−0.0039	0.3545	0.0577	0.0687	**0.523**	0	0	−0.3633	**1.0691**	0.2942	0.0096	0.2149	0.0527	**0.6473**	0.0754
District	Baoshan					Jiading					Jinshan				
*μ_i_*	*a* (I)	*b*_1_ (II)	*b*_2_ (III)	*b*_2_ (IV)	*c* (V)	*a* (I)	*b*_1_ (II)	*b*_2_ (III)	*b*_2_ (IV)	*c* (V)	*a* (I)	*b*_1_ (II)	*b*_2_ (III)	*b*_2_ (IV)	*c* (V)

*μ*_PP_	0	0.4626	0.5374	0	0	0.2985	0.7015	0	0	0	1	0	0	0	0
*μ*_GDPP_	0	0	−0.5463	1.5463	0	0	0.1629	0.8371	0	0	0	0.6623	0.3377	0	0
*μ*_IOUA_	0	0	−0.608	1.608	0	0	0	0	0	1	0.326	0.674	0	0	0
*μ*_BAR_	0	0.3538	0.6462	0	0	0	0.8576	0.1424	0	0	0.9665	0.0335	0	0	0
*μ*_GAR_	0	0	0	0	1	0	0.1728	0.8272	0	0	0.4627	0.5373	0	0	0
*μ*_WRP_	0	0	−0.9613	1.9613	0	0	0	−0.1093	1.1093	0	0	0	−0.9692	1.9692	0
*μ*_WRUR_	1	0	0	0	0	0	0.9	0.1	0	0	0	0.7	0.3	0	0
*μ*_STA_	0	1	0	0	0	1	0	0	0	0	1	0	0	0	0
*μ*	0.0724	0.1778	−0.1029	**0.7161**	0.1366	0.0964	0.3605	**0.2591**	0.1273	0.1567	0.4741	**0.3373**	−0.0373	0.2259	0
District	Songjiang					Qingpu					Fengxian				
*μ_i_*	*a* (I)	*b*_1_ (II)	*b*_2_ (III)	*b*_2_ (IV)	*c* (V)	*a* (I)	*b*_1_ (II)	*b*_2_ (III)	*b*_2_ (IV)	*c* (V)	*a* (I)	*b*_1_ (II)	*b*_2_ (III)	*b*_2_ (IV)	*c* (V)

*μ*_PP_	0.543	0.457	0	0	0	1	0	0	0	0	1	0	0	0	0
*μ*_GDPP_	0	0.3022	0.6978	0	0	0	0	0.2039	0.7961	0	0	0.3219	0.6781	0	0
*μ*_IOUA_	0	0	0.482	0.518	0	0.695	0.305	0	0	0	0.918	0.082	0	0	0
*μ*_BAR_	0	0.9244	0.0756	0	0	0.7487	0.2513	0	0	0	0.8508	0.1492	0	0	0
*μ*_GAR_	0	0	0.548	0.452	0	−0.638	1.638	0	0	0	0.3387	0.6613	0	0	0
*μ*_WRP_	0	0	0	0	1	0	0	−0.7197	1.7197	0	0	0	−0.974	1.974	0
*μ*_WRUR_	0	1	0	0	0	0.6	0.4	0	0	0	0	0.9	0.1	0	0
*μ*_STA_	1	0	0	0	0	0.6	0.4	0	0	0	1	0	0	0	0
*μ*	0.1362	0.3262	**0.292**	0.1431	0.0725	0.3722	**0.3586**	−0.0511	0.3203	0	**0.5321**	0.2411	0.0003	0.2265	0
District	Chongming														
*μ_i_*	*a* (I)	*b*_1_ (II)	*b*_2_ (III)	*b*_2_ (IV)	*c* (V)										

*μ*_PP_	1	0	0	0	0										
*μ*_GDPP_	1	0	0	0	0										
*μ*_IOUA_	1	0	0	0	0										
*μ*_BAR_	1	0	0	0	0										
*μ*_GAR_	0	0	0	0	1										
*μ*_WRP_	0	0	0	0	1										
*μ*_WRUR_	1	0	0	0	0										
*μ*_STA_	1	0	0	0	0										
*μ*	**0.7486**	0	0	0	0.2514

**Table 3:** Connection degree calibrated from comprehensive SPA.

District	Pudong					Huangpu					Xuhui				
*μ_i_*	I	II	III	IV	V	I	II	III	IV	V	I	II	III	IV	V
*μ*_PP_	−0.2725	1	0.2725	−1	−1	−1	−1	−1	−1	1	−1	−1	0.3567	1	−0.3567
*μ*_GDPP_	−1	−1	−1	−1	1	−1	−0.4783	1	0.4783	−1	−1	−1	0.2065	1	−0.2065
*μ*_IOUA_	−1	−1	−1	1	−0.163	−1	0.831	1	−0.831	−1	−1	−1	−0.911	1	0.911
*μ*_BAR_	−1	0.8626	1	−0.8626	−1	−1	−1	−1	−1	1	−1	−1	0.0467	1	−0.0467
*μ*_GAR_	−1	−1	−0.4635	1	0.4635	1	0.7873	−1	−1	−1	−1	−1	−1	−1	1
*μ*_WRP_	−1	−1	−1	−1	1	0.1805	1	−0.1805	−1	−1	−0.0893	1	0.0893	−1	−1
*μ*_WRUR_	−1	−1	−0.2	1	0.2	−1	−1	−1	−1	1	−1	−1	−1	1	1
*μ*_STA_	−1	−1	−1	−1	1	−1	−1	−0.8	1	0.8	−1	−1	−1	1	1
*μ*	−0.8813	−0.3991	−0.3628	−0.2377	**0.0610**	−0.5896	−0.1553	−0.2710	−0.6479	**−0.1394**	−0.8951	−0.7696	−0.2977	**0.4952**	0.1928
District	Changning					Jingan					Putuo				
*μ_i_*	I	II	III	IV	V	I	II	III	IV	V	I	II	III	IV	V

*μ*_PP_	−1	−1	0.6024	1	−0.6024	−1	−1	−0.8604	1	0.8604	−1	−1	−0.1183	1	0.1183
*μ*_GDPP_	−1	−1	0.0166	1	−0.0166	−1	−1	−0.5843	1	0.5843	−1	−1	0.2046	1	−0.2046
*μ*_IOUA_	0.078	1	−0.078	−1	−1	−0.632	1	0.632	−1	−1	0.148	1	−0.148	−1	−1
*μ*_BAR_	−1	−1	0.1913	1	−0.1913	−1	−1	−1	−1	1	−1	−1	0.1567	1	−0.1567
*μ*_GAR_	−1	−1	−1	−1	1	−1	−1	0.598	1	−0.598	−1	−1	−0.953	1	0.953
*μ*_WRP_	−0.359	1	0.359	−1	−1	1	−1	−1	−1	−1	−0.6858	1	0.6858	−1	−1
*μ*_WRUR_	−1	−1	−1	−1	1	−1	−1	−1	−1	1	−1	−1	−1	1	1
*μ*_STA_	−1	−1	−0.6	1	0.6	−1	−1	−0.8	1	0.8	−1	−1	−0.4	1	0.4
*μ*	−0.7565	−0.4548	−0.0843	**0.0276**	−0.1592	−0.7117	−0.6852	−0.4270	0.0071	**0.1387**	−0.7831	−0.4548	−0.1352	**0.4548**	−0.0817
District	Hongkou					Yangpu					Minhang				
*μ_i_*	I	II	III	IV	V	I	II	III	IV	V	I	II	III	IV	V

*μ*_PP_	−1	−1	−1	−1	1	−1	−1	0.1252	1	−0.1252	−1	0.8111	1	−0.8111	−1
*μ*_GDPP_	−1	−1	−0.8653	1	0.8653	−1	−1	0.5466	1	−0.5466	−1	−1	0.0381	1	−0.0381
*μ*_IOUA_	0.64	1	−0.64	−1	−1	−1	−1	−1	−1	1	−1	−1	−0.252	1	0.252
*μ*_BAR_	−1	−1	−1	−1	1	−1	−1	0.488	1	−0.488	−1	0.3814	1	−0.3814	−1
*μ*_GAR_	−0.7033	1	0.7033	−1	−1	−1	−1	0.4765	1	−0.4765	−1	−1	0.985	1	−0.985
*μ*_WRP_	−0.9087	1	0.9087	−1	−1	−1	−1	0.6405	1	−0.6405	−1	−1	−0.3135	1	0.3135
*μ*_WRUR_	−1	−1	−1	−1	1	−1	−1	−0.2	1	0.2	−1	−1	1	1	−1
*μ*_STA_	−1	−1	0	1	0	−1	−0.6	1	0.6	−1	0.2	1	−0.2	−1	−1
*μ*	−0.6906	−0.1804	−0.4208	−0.5936	**0.1114**	−1.0000	−0.9808	0.1917	**0.6660**	−0.1917	−0.9423	−0.4046	**0.4427**	0.4046	−0.5004
District	Baoshan					Jiading					Jinshan				
*μ_i_*	I	II	III	IV	V	I	II	III	IV	V	I	II	III	IV	V

*μ*_PP_	−1	0.6682	1	−0.6682	−1	0.2985	1	−0.2985	−1	−1	1	−1	−1	−1	−1
*μ*_GDPP_	−1	−1	0.4537	1	−0.4537	−1	0.3257	1	−0.3257	−1	−0.6754	1	0.6754	−1	−1
*μ*_IOUA_	−1	−1	0.696	1	−0.696	−1	−1	−1	−1	1	0.353	1	−0.353	−1	−1
*μ*_BAR_	0.4246	1	−0.4246	−1	−1	−0.8547	1	0.8547	−1	−1	0.9665	1	−0.9665	−1	−1
*μ*_GAR_	−1	−1	−1	−1	1	−1	0.432	1	−0.432	−1	0.4627	1	−0.4627	−1	−1
*μ*_WRP_	−1	−1	0.0388	1	−0.0388	−1	−1	0.8908	1	−0.8908	−1	−1	0.0308	1	−0.0308
*μ*_WRUR_	1	−1	−1	−1	−1	−1	0	1	0	−1	−1	0	1	0	−1
*μ*_STA_	0	1	0	−1	−1	1	−1	−1	−1	−1	1	−1	−1	−1	−1
*μ*	−0.5890	−0.3367	**0.0713**	−0.0905	−0.4823	−0.6705	0.0997	**0.3431**	−0.5107	−0.6726	0.1764	**0.2707**	−0.2881	−0.6932	−0.8884
District	Songjiang					Qingpu					Fengxian				
*μ_i_*	I	II	III	IV	V	I	II	III	IV	V	I	II	III	IV	V

*μ*_PP_	0.543	1	−0.543	−1	−1	1	−1	−1	−1	−1	1	−1	−1	−1	−1
*μ*_GDPP_	−1	0.6043	1	−0.6043	−1	−1	−0.7961	1	0.7961	−1	−1	0.6438	1	−0.6438	−1
*μ*_IOUA_	−1	−0.518	1	0.518	−1	0.695	1	−0.695	−1	−1	0.918	1	−0.918	−1	−1
*μ*_BAR_	−0.4535	1	0.4535	−1	−1	0.7487	1	−0.7487	−1	−1	0.8508	1	−0.8508	−1	−1
*μ*_GAR_	−1	−0.452	1	0.452	−1	−0.638	1	0.638	−1	−1	0.3387	1	−0.3387	−1	−1
*μ*_WRP_	−1	−1	−1	−1	1	−1	0.2803	1	−0.2803	−1	−1	−1	0.026	1	−0.026
*μ*_WRUR_	−0.3333	1	0.3333	−1	−1	−1	1	1	−1	−1	−1	0	1	0	−1
*μ*_STA_	1	−1	−1	−1	−1	0.6	1	−0.6	−1	−1	−1	−1	−1	−1	1
*μ*	−0.5205	0.1739	**0.2901**	−0.5005	−0.7696	−0.0224	**0.3122**	0.0224	−0.6385	−1.0000	0.0847	**0.2154**	**−**0.2932	−0.6380	−0.7916
District	Chongming														
*μ_i_*	I	II	III	IV	V										

*μ*_PP_	1	−1	−1	−1	−1										
*μ*_GDPP_	1	−1	−1	−1	−1										
*μ*_IOUA_	1	−1	−1	−1	−1										
*μ*_BAR_	1	−1	−1	−1	−1										
*μ*_GAR_	−1	−1	−1	−1	1										
*μ*_WRP_	−1	−1	−1	−1	1										
*μ*_WRUR_	1	1	−1	−1	−1										
*μ*_STA_	1	−1	−1	−1	−1										
*μ*	**0.4952**	−0.8472	−1.0000	−1.0000	−0.4952

**Table 4:** Connection degree calibrated from proposed IFN-SPA.

District	Pudong					Huangpu					Xuhui				
*μ_i_*	I	II	III	IV	V	I	II	III	IV	V	I	II	III	IV	V
*μ*_PP_	0	1	0.2725	0	0	0	0	0	0.8619	1	0	0	0.3567	1	0
*μ*_GDPP_	0	0	0	0	1	0	0	1	0.4783	0	0	0	0.2065	1	0
*μ*_IOUA_	0	0	0.1635	1	0	0	0.4155	1	0	0	0	0	0	1	0.4555
*μ*_BAR_	0	0.8626	1	0	0	0	0	0	0.0392	1	0	0	0.0467	1	0
*μ*_GAR_	0	0	0	1	0.4635	1	0.7873	0	0	0	0	0	0	0.844	1
*μ*_WRP_	0	0	0	0.631	1	0.1783	1	0	0	0	0	1	0.0893	0	0
*μ*_WRUR_	0	0	0	1	0.2	0	0	0	0	1	0	0	0	1	1
*μ*_STA_	0	0	0	0.6	1	0	0	0	1	0.8	0	0	0	1	1
*μ*	0.0000	0.2903	0.2176	**0.4726**	0.3973	0.1577	0.2886	0.3125	0.2687	**0.4255**	0.0000	0.1152	0.1074	**0.8634**	0.3334
District	Changning					Jingan					Putuo				
*μ_i_*	I	II	III	IV	V	I	II	III	IV	V	I	II	III	IV	V

*μ*_PP_	0	0	0.6024	1	0	0	0	0	1	0.8605	0	0	0	1	0.1183
*μ*_GDPP_	0	0	0.0166	1	0	0	0	0	1	0.5843	0	0	0.2046	1	0
*μ*_IOUA_	0.078	1	0	0	0	0	1	0.632	0	0	0.148	1	0	0	0
*μ*_BAR_	0	0	0.1913	1	0	0	0	0	0.8761	1	0	0	0.1566	1	0
*μ*_GAR_	0	0	0	0	1	0	0	0.598	1	0	0	0	0	1	0.953
*μ*_WRP_	0	1	0.359	0	0	1	0.8267	0	0	0	0	1	0.6858	0	0
*μ*_WRUR_	0	0	0	0.2	1	0	0	0	0	1	0	0	0	1	1
*μ*_STA_	0	0	0	1	0.6	0	0	0	1	0.8	0	0	0	1	0.4
*μ*	0.0123	0.2726	0.1704	**0.5291**	0.2425	0.1152	0.2526	0.1815	**0.6327**	0.4934	0.0233	0.2726	0.1338	**0.7274**	0.2457
District	Hongkou					Yangpu					Minhang				
*μ_i_*	I	II	III	IV	V	I	II	III	IV	V	I	II	III	IV	V

*μ*_PP_	0	0	0	0.4248	1	0	0	0.1252	1	0	0	0.8111	1	0	0
*μ*_GDPP_	0	0	0	1	0.8653	0	0	0.5466	1	0	0	0	0.0381	1	0
*μ*_IOUA_	0.64	1	0	0	0	0	0	0	0	1	0	0	0	1	0.126
*μ*_BAR_	0	0	0	0.8526	1	0	0	0.4888	1	0	0	0.3814	1	0	0
*μ*_GAR_	0	1	0.7033	0	0	0	0	0	1	0.4765	0	0	0.958	1	0
*μ*_WRP_	0	1	0.9088	0	0	0	0	0.6405	1	0	0	0	0	1	0.3135
*μ*_WRUR_	0	0	0	0	1	0	0	0	1	0.2	0	0	1	1	0
*μ*_STA_	0	0	0	1	0	0	0	1	0.6	0	0.2	1	0	0	0
*μ*	0.1007	0.4098	0.2012	**0.3982**	0.5212	0.0000	0.0000	0.2992	**0.8234**	0.2381	0.0096	0.2367	0.5244	**0.6413**	0.0559
District	Baoshan					Jiading					Jinshan				
*μ_i_*	I	II	III	IV	V	I	II	III	IV	V	I	II	III	IV	V

*μ*_PP_	0	0.6682	1	0	0	0.2985	1	0	0	0	1	0.374	0	0	0
*μ*_GDPP_	0	0	0.4537	1	0	0	0.3257	1	0	0	0	1	0.6754	0	0
*μ*_IOUA_	0	0	0.696	1	0	0	0	0	0.229	1	0.353	1	0	0	0
*μ*_BAR_	0	0.4246	1	0	0	0	1	0.8645	0	0	0.9665	1	0	0	0
*μ*_GAR_	0	0	0	0.4355	1	0	0.432	1	0	0	0.4627	1	0	0	0
*μ*_WRP_	0	0	0.0388	1	0	0	0	0.8908	1	0	0	0	0.0308	1	0
*μ*_WRUR_	1	0.6923	0	0	0	0	0	1	0	0	0	0	1	0.5	0
*μ*_STA_	0	1	0	0	0	1	0.4	0	0	0	1	0	0	0	0
*μ*	0.0764	0.2726	**0.4950**	0.4874	0.1372	0.0968	0.4396	**0.5988**	0.1512	0.1574	0.4728	**0.6581**	0.1847	0.1534	0.0000
District	Songjiang					Qingpu					Fengxian				
*μ_i_*	I	II	III	IV	V	I	II	III	IV	V	I	II	III	IV	V

*μ*_PP_	0.543	1	0	0	0	1	0.813	0	0	0	1	0.698	0	0	0
*μ*_GDPP_	0	0.6043	1	0	0	0	0	1	0.7961	0	0	0.6438	1	0	0
*μ*_IOUA_	0	0	1	0.518	0	0.695	1	0	0	0	0.918	1	0	0	0
*μ*_BAR_	0	1	0.4535	0	0	0.7487	1	0	0	0	0.8568	1	0	0	0
*μ*_GAR_	0	0	1	0.452	0	0	1	0.638	0	0	0.3387	1	0	0	0
*μ*_WRP_	0	0	0	0.6045	1	0	0	0.2803	1	0	0	0	0.026	1	0
*μ*_WRUR_	0	1	0.3333	0	0	0	1	1	0	0	0	0	1	0	0
*μ*_STA_	1	0	0	0	0	0.6	1	0	0	0	1	0.4	0	0	0
*μ*	0.1367	0.4807	**0.5420**	0.2132	0.1152	0.4118	**0.6992**	0.3513	0.2387	0.0000	0.5285	**0.6750**	0.2345	0.1152	0.0000
District	Chongming														
*μ_i_*	I	II	III	IV	V										

*μ*_PP_	1	0	0	0	0										
*μ*_GDPP_	1	0.7625	0	0	0										
*μ*_IOUA_	1	0	0	0	0										
*μ*_BAR_	1	0	0	0	0										
*μ*_GAR_	0	0	0	0.0185	1										
*μ*_WRP_	0	0	0	0.7823	1										
*μ*_WRUR_	1	1	0	0	0										
*μ*_STA_	1	0	0	0	0										
*μ*	**0.7476**	0.1947	0.0000	0.0927	0.2524										

## Declaration of Competing Interest

The authors declare that they have no known competing financial interests or personal relationships that could have appeared to influence the work reported in this paper.

## Appendix Supplementary materials

Image, application 1

## References

[1] Lyu H.M., Shen S.L., Zhou A.N. (2021). The development of IFN-SPA: a new risk assessment method of urban water quality and its application in Shanghai. J. Clean. Prod..

[2] Wang W.S., Jin J.L., Ding J., Li Y.Q. (2009). A new approach to water resources system assessment: set pair analysis method. Sci. China.

[3] Zhao K.Q., Huang D.C., Lu Y.Z. (2000). A new network planning method based on the connection number a+bi+cj. Syst. Eng. Electron..

[4] Zou Q., Song L., Guo J. (2013). Comprehensive flood risk assessment based on set pair analysis-variable fuzzy sets model and fuzzy AHP. Stoch. Environ. Res. Risk Assess..

[5] Lyu H.M., Shen S.L., Yang J., Yin Z.Y. (2019). Inundation analysis of metro systems with the storm water management model incorporated into a geographical information system: a case study in Shanghai. Hydrol. Earth Syst. Sci..

[6] Lin S.S., Shen S.L., Zhou A., Xu Y.S. (2020). Approach based on TOPSIS and Monte Carlo simulation methods to evaluate lake eutrophication levels. Water Res..

[7] Lyu H.M., Sun W.J., Shen S.L., Zhou A. (2020). Risk assessment using a new consulting process in fuzzy AHP. J. Constr. Eng. Manag. ASCE.

[8] Lyu H.M., Shen S.L., Yang J., Zhou A. (2020). Risk assessment of earthquake-triggered geohazards surrounding Wenchuan, China. Nat. Hazard. Rev. ASCE.

[9] Lyu H.M., Zhou W.H., Shen S.L., Zhou A.N. (2020). Inundation risk assessment of metro system using AHP and TFN-AHP in Shenzhen. Sustain. Cities Soc..

[10] Lyu H.M., Shen S.L., Zhou A.N., Yang J. (2020). Risk assessment of mega-city infrastructures related to land subsidence using improved trapezoidal FAHP. Sci. Total Environ..

[11] Saaty T.L. (1977). A scaling method for priorities in hierarchical structures. J. Math. Psychol..

